# High prevalence of SARS-CoV-2 antibodies among frontline healthcare workers during the COVID-19 pandemic in three states of Nigeria from August to December 2022

**DOI:** 10.1371/journal.pone.0354606

**Published:** 2026-08-10

**Authors:** Nnaemeka C. Iriemenam, Sophia Osawe, Olusola A. Akanbi, Oluwaseun Akinmulero, Ado G. Abubakar, Mary Okoli, Rita Okonkwo, Oyewole Oyedele, Nifarta Andrew, Olumide Okunoye, Ugochi Ezenwelu, Asmau Aminu-Alhaji, Victor Ejeh, Martin Edun, Edward Kombu, Ndidi Agala, Diana Martin, Alash’le Abimiku, McPaul I. Okoye, SECURE Nigeria Project Team

**Affiliations:** 1 Division of Global HIV and TB, U.S. Centers for Disease Control and Prevention, Abuja, Federal Capital Territory, Nigeria; 2 International Research Centre of Excellence, Institute of Human Virology, Abuja, Federal Capital Territory, Nigeria; 3 Nigeria Centre for Disease Control and Prevention, Abuja, Federal Capital Territory, Nigeria; 4 Division of Global Health Protection, U.S. Center for Disease Control and Prevention, Abuja, Federal Capital Territory, Nigeria; 5 AIDS Prevention Initiatives in Nigeria, Abuja, Federal Capital Territory, Nigeria; 6 Division of Parasitic Diseases and Malaria, U.S. Centers for Disease Control and Prevention, Atlanta, Georgia, United States of America; Federal University of Rio de Janeiro, BRAZIL

## Abstract

**Background:**

Frontline healthcare workers (HCWs) are among the first professionals significantly impacted by epidemics and pandemics. During the COVID-19 pandemic, HCWs had an increased risk of infection. This study aimed to determine the seroprevalence of severe acute respiratory syndrome coronavirus 2 (SARS-CoV-2) among Nigerian HCWs.

**Methods:**

A cross-sectional study of 1,247 randomly selected HCWs, was conducted across seven healthcare facilities in Nigeria, from August 2022 through December 2022 (Epi week 31−51). This study had two parts: the knowledge, attitude, and practices (KAP) survey and the seroprevalence of SARS-CoV-2 antibodies. Healthcare cadres enrolled were nurses, laboratorians, doctors, and pharmacists who provided informed consent before enrollment. An antigen-based COVID-19 rapid diagnostic test was used to screen all HCWs at enrollment, and a SARS-CoV-2 Multi-Antigen IgG assay was used to test for the presence of SARS-CoV-2 antibodies for the seroprevalence component of the study.

**Findings:**

A total of 1,247 HCWs were enrolled 682 (54.6%) nurses, 286 (22.9%) laboratorians, 222(18.0%) doctors, and 57(4.5%) pharmacists. The majority HCWs were males (70.5%), lived in urban areas (90.2%), had tertiary education (64.0%), and were married (81.7%). The proportion of HCWs that used drugs or smoked was 0.2%. The seropositivity of SARS-CoV-2 amongst HCWs was 98.2% of which 80.6% where due to the Nucleocapsid protein and 17.6% due to the Spike protein. Nurses were three times more likely to be seropositive for COVID-19 than laboratorians (*P < 0.05*).

**Interpretation:**

Our findings reveal a high prevalence of SARS-CoV-2 antibodies among HCWs, especially among nurses. At the time of this study, half of the HCWs were unvaccinated for COVID-19. As frontline HCWs remain at increased risk of occupational exposure, this study highlights the need to strengthen infection prevention strategies, particularly vaccination and infection prevention and control measures, to protect the healthcare workforce.

## Introduction

The coronavirus disease 2019 (COVID-19) pandemic, caused by severe acute respiratory syndrome coronavirus 2 (SARS-CoV-2), had a profound impact on global health and well-being, overwhelming healthcare systems and disrupting the delivery of essential health services worldwide. [1]. A socio-economic analysis in Nigeria reported that the COVID-19 pandemic affected the healthcare system and many socio-economic activities, resulting in an economic downturn [2]. The pandemic had a particularly huge impact on routine healthcare services as resources were redirected to control the surge of the virus across the population. As a result, management and care for other diseases were negatively impacted leading to delays in treatment and poor delivery of quality healthcare services [3]. The COVID-19 pandemic highlighted the need to ensure adaptable health systems that can absorb the demands in managing disease outbreaks while protecting the health and wellbeing of the healthcare workforce.

Healthcare workers (HCWs) are at the forefront of public health emergency response, providing care for the earliest cases of emerging infectious diseases. This frontline role places them at a significantly increased risk of occupational exposure to infectious pathogens. [4]. The United States Centers for Disease Control and Prevention (U.S. CDC) reported that 315,531 COVID-19 cases were reported between February and April 2020 in the United States of which 49,370 included information on whether patients were HCWs and 19% of these cases were HCWs [5]. With the high risk of COVID-19 infections among HCWs, tools were adopted to assess risk factors for the disease [6–8]. The knowledge, attitudes, and practices (KAP) model was adopted to collect information about HCWs [8]. KAP scores inform behaviors and measure the understanding and adherence to safety precautions among HCWs during pandemics like COVID-19. Although numerous studies have been conducted on the KAP of COVID-19 among HCWs, there is limited evidence about the relationship between KAP and the seroprevalence of COVID-19 among HCWs [9–12].

The COVID-19 vaccine protects against severe disease and has been recommended to general populations from > 6 months of age [13]. As of 26^th^ November 2023, 13,516,282,548 COVID-19 vaccine doses were administered globally, of which 67% of the global population have been vaccinated with a complete primary series and only 32% with at least one booster dose [1]. Although COVID-19 vaccines were introduced and recommended for healthcare workers (HCWs) in Nigeria in March 2021, a study conducted between April and June 2022 found that only 44% of HCWs had received at least one dose of a COVID-19 vaccine, either partially or fully completing the vaccination schedule.[14]. Vaccine hesitancy amongst HCWs increases the risk of disease transmission within the healthcare setting and across the population.

While studies have been conducted in Nigeria and have reported a seroprevalence of SARS-CoV-2 between 9.3 and 94.0% among HCWs, most of these studies were conducted earlier, between the onset of the pandemic and 2021, or on smaller sample sizes [14–17]. Estimating SARS-CoV-2 seroprevalence among HCWs is critical for understanding the level of exposure and immunity which can be used to guide strategies to promote disease control amongst HCWs. This study aims to estimate SARS-CoV-2 seroprevalence among different cadres of HCWs during the fourth wave of COVID-19.

## Methods

### Study design

A multicenter cross-sectional study was conducted in seven health facilities in three Nigerian states (Plateau, Oyo, and Rivers), between August 2022 and December 2022 (Epi week 31–51). In collaboration with the U.S. CDC and the Nigerian Centre for Disease Control and Prevention (NCDC), these three states were purposively selected to represent the states that studies on seroprevalence were not conducted. Secondary and tertiary healthcare facilities were allocated sample quotas based on their pool of HCWs and according to probability proportional to size. The study utilized a state-stratified random sampling strategy to generate a line list of all HCWs at each selected health facility within the state.

### Study participant

Frontline HCWs, including medical doctors, laboratorians, nurses, and pharmacists from the selected healthcare facilities, were randomly selected to participate in the study. Using a randomized line list of HCWs, every fourth name was consecutively invited to participate in the study. Invitations were sent via text messages and phone calls to the selected HCWs. Using a precision-based approach, the sample size estimation was 1,247 HCWs assuming a seroprevalence of 15%, 95% confidence level, precision level of ±3%, design effect of 1.5, projected 30% non-response rate, medium effect size of 10%, statistical power of 80% and a type 1 error rate of 5% for the comparison of prevalence estimates across the cadres of HCWs. A structured self-administered questionnaire designed on the Research Electronic Data Capture (REDCap) application was used to collect information on KAP to COVID-19, socio-demographic, epidemiological information (exposure, clinical and health-related characteristics) and COVID-19 vaccination data, from each participant.

### Procedure

Five milliliters of venous blood were collected in EDTA tubes at designated phlebotomy sections by trained phlebotomists and transported to the processing laboratories in cold chain (2-8^o^C), at each selected health facility Whole blood samples were processed into plasma within four hours of sample collection, by centrifugation at 2,200−2,500 resolutions per minute for 20 minutes.. The plasma samples were separated, aliquoted into two 1 milliliter cryovials, and stored in a -20^o^C freezer until transported to the National Reference Laboratory (NRL) for testing. Shipments were temperature-controlled using Credo Cubes (M50 series) shipping container, through an authorized local courier provider. Additionally, nasopharyngeal swabs were collected for antigen-based rapid diagnostic test (RDT) COVID-19 testing (SD Biosensor COVID-19 Rapid Antigen Test Kit), following the manufacturer’s instructions [18]. Participants who tested positive by the SARS-CoV-2 rapid antigen test were referred for confirmatory testing using reverse transcription polymerase chain reaction (RT-PCR). [19].

### SARS-CoV-2 Multi-Antigen IgG Bead Assay (SARS2MBA)

The SARS2MBA developed by the U.S. CDC was validated in Nigeria before use and described in previous studies [20,21]. Briefly, plasma samples were diluted to 1:400 in Buffer B (1X phosphate-buffered saline [PBS], 0.5% casein, 0.5% polyvinyl alcohol [PVA], 0.8% polyvinylpyrrolidone [PVP], 0.3% Tween-20, 0.02% sodium azide (NaN3) and 3 µg/ml Escherichia coli extract). The diluted sample (50 µl) was incubated with beads (1250 µl/well/antigen) in a 96-well flat bottom (Bio-Plex assay) plate for 1.5 hours. The beads were washed three times with 200 µl phosphate-buffered saline solution (PBST) (1X PBS + 0.05% Tween-20) using a magnetic 96-well separator (Thermofisher) to hold the beads to the plate while the liquid was decanted. They were incubated with 50 ng IgG (Southern Biotech, Birmingham, AL) and 20 ng IgG4 (Southern Biotech) to detect bound antibodies for 45 minutes, after which they were washed three times with PBST and incubated with 250 ng SAPE (Invitrogen, San Francisco, CA) for 30 minutes. The beads were washed three times with PBST and incubated with 1X PBS, 0.5% bovine serum albumin (BSA), 0.05% Tween-20, and 0.02% NaN3 for 30 minutes to remove any loosely bound antibodies. The beads were rewashed, suspended in 1X PBS, and stored overnight at 4°C. The next day, the beads were read on a Luminex MAGPIX® instrument with the average median fluorescence intensity (MFI) determined for each sample and seropositivity to SARS-CoV-2 antibodies decided if samples tested positive for the spike glycoprotein (S) and receptor-binding domain 591 (RBD591) within the spike glycoprotein.

### Result interpretation for SARS-CoV-2 seropositivity

Antibodies against SARS-CoV-2 were previously established based on the validated U.S. CDC-established MFI cut-off values: S (125), RBD591 (971), and N proteins (1199), respectively. SARS-CoV-2 seropositivity was based on set median fluorescence intensity (MFI) cut off values for S, RBD591 and N proteins. Samples were reported as seronegative for SARS-CoV-2 if both S and RBD591 had cut-off MFI values were below the positivity threshold (S-/RBD591+ or S + /RBD591-), irrespective of the N values (N + /N-). SARS-CoV-2 seropositivity was defined as IgG antibody positive for both S and N proteins (S + N+). Additionally, current SARS-CoV-2 seropositivity was defined as a combination of S + /RBD591 + /N + . However, antibody positivity to S and RBD591 protein and negative to N protein could have four different interpretations: [1] the participants were vaccinated, [2] the participants with no natural infection or a natural infection with waning N antibodies, [3] the clients have antibodies from SARS-CoV-2 infection and failed to develop detectable N antibodies, or [4] a combination of antibodies from SARS-CoV-2 infection and vaccination (hybrid immunity).

### Study instrument

A structured interviewer administered questionnaire was used to assess the knowledge, attitude, and practice (KAP) of HCWs towards COVID-19. The knowledge section had 24 questions assessing the awareness of the etiology, symptoms, transmission, prevention, control and management of COVID-19. Attitude section included nine questions assessing perceptions of HCWs about infection prevention and control (IPC), management of cases, mitigating strategies, and incentives in a 5 points Likert scale, and a 3 points scale for only two questions. The practice section included six questions assessing the use of personal protective equipment, adherence to IPC, individual treatment of flu-like symptoms in a 7 points scale, and 4 points scale for only one question. Good KAP towards COVID-19 was derived from the total score over the total obtainable score for each KAP multiplied by 100 (**S1 Form**).

### Study variables

The primary outcome for this study was seropositivity of SARS-CoV-2 among HCWs. The characteristics of HCWs were further grouped into five sections, including the socio-demographic characteristics, household and facility information, exposure, clinical and health-related information, and lifestyle factors among the study participants. Exposure characteristics assessed include COVID-19 testing history, vaccination, self-reported diagnosis, and symptoms, exposure, travel history. Lifestyle characteristics like body mass index (BMI), smoking, alcohol, drug use, knowledge, attitude, and practices towards COVID-19 prevention and control were also evaluated. Occupational exposure was defined as contact with positive patients or infected sample. Also, good knowledge, attitude, and practice (KAP) towards COVID-19 was defined by ≥80% score to the KAP questions.

### Statistical analysis

Data from RedCap was exported to Microsoft Excel version 365 for data cleaning and coding. Cleaned and coded data was then imported into the statistical software STATA (version 18) for statistical analysis. Descriptive analysis was presented in frequencies (percentages) for all the categorical variables and the mean (standard deviation) or equivalent median (interquartile range) for continuous variables. Normality was assessed via the Shapiro-Wilk test for anthropometric data (body mass index), and a graphical presentation (histogram) was reported. Socio-demographic characteristics, household and facility information, exposure, clinical and health-related information, and lifestyle factors were five classes of explanatory variables built-in models of five blocks, with each class constituting a block to identify factors associated with the seropositivity rate (due to current, previous and all infection) in a stepwise model selection approach. The block of model selection was performed based on the variable importance due to the significant change in the model likelihood ruled at *p < 0.20* at the univariate level.

The 3-series of multivariable model analysis was conducted via the log-binomial model to evaluate the adjusted variable effect on the 3-distinct seropositivity outcome, respectively, and the variables identified from the five model blocks made up the predictors. The model block that minimizes information loss was determined from the minimum Akaike information criterion (AIC) statistics. The level of association and significance of the SARS-CoV-2 IgG positivity with the identified predictors was assessed, and the corresponding adjusted and crude risk ratio and the 95% confidence interval were reported at a 5% alpha significance level.

### Ethical statement

Ethical approvals were received from the National Health Research Ethics Committee (NHREC/01/01/2007) and the institutional ethical committees at each healthcare facility. This activity was reviewed by the U.S. CDC and deemed not research. It was conducted consistent with applicable federal law and U.S. CDC policy.§. U.S. CDC staff did not contact participants nor access personal identifying information. Prior to sample collection, eligible HCWs in selected healthcare facilities were verbally recruited to participate in the survey. Interested HCWs were provided with adequate information on the study objectives, procedure and potential risk from participating in the study and were expected to sign an informed consent form. Participants that signed the informed consent form were included and data was deidentified to protect the confidentiality of the study participants. Also, participation in the study was completely voluntary and participants were allowed to withdraw from the study at any period they felt uncomfortable.

§ See, e.g., 45 C.F.R. part 46.102(l) [2], 21 C.F.R. part 56; 42 U.S.C. §241(d); 5 U.S.C. §552a; 44 U.S.C. §3501 et seq.

## Results

### Descriptive statistics of HCWs in the three Nigerian States

A total of 1,247 HCWs were enrolled, with 682 (54.6%) nurses and 56 (4.5%) pharmacists which were the least represented HCW group. The majority of the Majority of the doctors (65.8%) and pharmacists (60.7%) were female while nurses (90%) and laboratorians (60.7%) were male in majority. A total of 580 (46.6%) healthcare workers (HCWs) reported occupational exposure to COVID-19. Additionally, 40.9% had contact with suspected COVID-19 cases, while 36.8% reported contact with confirmed cases. Only 26 (2.1%) HCWs reported living with an individual suspected of having COVID-19.. Only 323 (26%) of the HCWs self-reported that they experienced symptoms of COVID-19, and 187 (15%) were previously diagnosed with COVID-19 infection. As part of the project, HCWs were tested for a current COVID-19 infection using RDT, and six (0.5%) tested positive (Table 1). Although the majority of the HCWs reported good knowledge (64.7%) and attitude (51.3%) about COVID-19 prevention and control measures, only 66 (5.3%) had good practices regarding COVID-19 infection prevention and control protocols. Laboratorians made up the highest proportion of HCWs with poor knowledge (40.9%), attitude (54.2%), and practice (97.9%) regarding COVID-19 prevention and control measures (Table 1).

**Table 1:** Characteristics of HCWs by Cadre in Three Nigerian States.

HCWs Characteristics	Overall N = 1247 (100.0%)	Cadre of Healthcare Workers			
		Doctor n = 222 (18.0%)	Laboratorian n = 286 (22.9%)	Nurse n = 682 (54.6%)	Pharmacist n = 57 (4.5%)
***Demographic***					
**Age**					
21-30 years	86 (6.8)	5 (2.3)	35 (12.3)	30 (4.4)	15 (26.7)
31-40 years	443 (35.5)	139 (62.6)	111 (38.8)	178 (26.1)	15 (26.7)
41-50 years	367 (29.5)	58 (26.1)	94 (32.8)	205 (30.1)	10 (18.0)
51-60 years	334 (26.8)	20 (9.0)	43 (15.0)	257 (37.6)	14 (25.0)
>60 years	17 (1.4)	0 (0.0)	3 (1.1)	12 (1.8)	2 (3.6)
**Sex**					
Male	878 (70.5)	76 (34.2)	166 (58.0)	614 (90.0)	22 (39.3)
Female	368 (29.5)	146 (65.8)	120 (42.0)	68 (10.0)	34 (60.7)
**Place of residence**					
Rural	35 (2.8)	3 (1.4)	7 (2.5)	24 (3.5)	1 (1.8)
Semi-urban	86 (7.0)	18 (8.1)	20 (6.9)	44 (6.5)	4 (7.1)
Urban	1125 (90.2)	201 (90.5)	259 (90.6)	614 (90.0)	51 (91.1)
**State of residence**					
Oyo	428 (34.4)	72 (32.4)	99 (34.6)	237 (34.8)	20 (35.7)
Plateau	430 (34.5)	83 (37.4)	97 (33.9)	233 (34.1)	17 (30.3)
Rivers	388 (31.1)	67 (30.2)	90 (31.5)	212 (31.1)	19 (34.0)
**Education**					
Postgraduate	449 (36.0)	141 (63.5)	98 (34.3)	192 (28.2)	18 (32.1)
Tertiary	797 (64.0)	81 (36.5)	188 (65.7)	490 (71.8)	38 (67.9)
**Marital status**					
Single	168 (13.5)	32 (14.4)	61 (21.3)	59 (8.7)	16 (28.6)
Married	1018 (81.7)	287 (84.2)	219 (76.6)	575 (84.3)	37 (66.10)
Separated	15 (1.2)	1 (0.5)	2 (0.7)	11 (1.6)	1 (1.8)
Widowed	45 (3.6)	2 (0.9)	4 (1.4)	37 (5.4)	2 (3.6)
**Religion**					
Christianity	1186 (95.2)	210 (94.5)	273 (95.4)	651 (95.4)	52 (92.9)
Islam	57 (4.6)	11 (5.0)	13 (4.6)	29 (4.6)	4 (7.1)
Traditional religion	3 (0.2)	1 (0.5)	0 (0.0)	2 (0.0)	0 (0.0)
***Facility/Household***					
**Facility of practice**					
BUTH	97 (7.8)	19 (8.6)	17 (5.9)	56 (8.9)	5 (9.0)
JUTH	217 (17.4)	41 (18.4)	50 (17.5)	119 (12.5)	7 (13.0)
LTH	97 (7.8)	18 (8.1)	22 (7.7)	53 (7.1)	4 (7.1)
PSSH	116 (9.3)	22 (9.9)	30 (10.5)	59 (8.7)	5 (8.9)
RSUTH	141 (11.3)	25 (11.3)	35 (12.2)	75 (11.0)	6 (10.7)
UCH	330 (26.4)	54 (24.3)	77 (26.9)	183 (26.8)	16 (28.0)
UPTH	248 (20.0)	43 (19.4)	55 (19.2)	137 (20.0)	13 (23.2)
**Ownership of facility**					
Public	1149 (92.2)	203 (91.4)	269 (94.1)	626 (91.8)	51 (91.1)
Private	97 (7.8)	19 (8.6)	17 (5.9)	56 (8.2)	5 (8.9)
**Years of practice**					
< 2years	43 (3.5)	0 (0.0)	30 (10.5)	3 (0.4)	10 (17.7)
2 - 5years	114 (9.2)	21 (9.5)	41 (14.3)	42 (6.2)	10 (17.7)
6 - 10years	256 (20.5)	81 (36.5)	56 (19.6)	111 (16.3)	8 (14.3)
11-15years	333 (26.7)	72 (32.4)	67 (23.4)	189 (27.7)	5 (8.9)
>15 years	500 (40.1)	48 (21.6)	92 (32.1)	337 (49.4)	23 (41.0)
**Household type**					
Self-contain	93 (7.5)	8 (3.6)	32 (11.2)	41 (6.0)	12 (21.4)
1 bedroom	102 (8.2)	25 (11.3)	30 (10.5)	44 (6.5)	3 (5.4)
2 bedrooms	303 (24.3)	67 (30.2)	61 (21.3)	157 (23.0)	18 (32.1)
3 bedrooms	504 (46.8)	97 (43.6)	127 (44.4)	345 (50.6)	15 (26.8)
Other	164 (13.2)	25 (11.3)	36 (12.6)	95 (13.9)	8 (14.3)
**Household size**					
1	89 (7.1)	29 (13.1)	25 (9.0)	30 (4.4)	5 (8.9)
2	92 (7.4)	19 (8.6)	29 (10.1)	40 (5.9)	4 (7.0)
3	137 (11.0)	28 (12.6)	26 (9.1)	78 (11.4)	5 (8.9)
4	257 (20.6)	56 (25.2)	55 (19.2)	138 (20.2)	8 (14.2)
5+	671 (53.9)	90 (40.5)	151 (52.8)	396 (58.1)	34 (61.0)
***Exposure***					
**Occupational exposure**					
Unexposed	666 (53.4)	122 (54.9)	159 (55.6)	349 (51.2)	36 (64.3)
Exposed	580 (46.6)	100 (45.1)	127 (44.4)	333 (48.8)	20 (35.7)
**COVID-19 vaccination** **status**					
Unvaccinated	624 (50.1)	78 (35.1)	199 (69.6)	321 (47.1)	26 (46.4)
Vaccinated	622 (49.9)	144 (64.9)	87 (30.4)	361 (52.9)	30 (53.4)
**Recent travel history**					
No	1225 (98.3)	215 (96.8)	278 (97.2)	679 (99.6)	53 (94.6)
Yes	21 (1.7)	7 (3.2)	8 (2.8)	3 (0.4)	3 (5.4)
**Attended a gathering of more than 50 persons**					
No	441 (35.4)	81 (36.5)	95 (33.2)	245 (35.9)	20 (35.7)
Yes	805 (64.6)	141 (63.5)	191 (66.8)	437 (64.1)	36 (64.3)
**Contact with a person with suspected COVID-19 infection**					
No	735 (59.0)	102 (45.9)	187 (64.9)	405 (59.4)	42 (75.0)
Yes	511 (40.9)	120 (54.1)	101 (35.0)	277 (40.6)	14 (25.0)
**Contact with a person with a confirmed COVID-19 infection**					
No	787 (63.2)	110(49.6)	200 (69.9)	433 (63.5)	44 (78.6)
Yes	459 (36.8)	112 (50.4)	86 (30.1)	249 (36.5)	12 (21.4)
**Live with a person with suspected COVID-19 infection**					
No	1220 (97.9)	217 (97.7)	278 (97.2)	672 (98.5)	53 (94.6)
Yes	26 (2.1)	5 (2.3)	8 (2.8)	10 (1.5)	3 (5.4)
***Clinical & Health-related***					
**Blood group**					
A	290 (23.3)	40 (18.0)	71 (24.8)	168 (24.6)	11 (19.6)
B	87 (7.0)	17 (7.7)	10 (3.5)	58 (8.5)	2 (3.6)
AB	241 (19.3)	35 (15.8)	60 (21.0)	137 (20.1)	9 (16.1)
O	628 (50.4)	130 (58.5)	145 (50.7)	319 (46.8)	34 (60.7)
**Ever been tested for COVID-19**					
No	656 (52.6)	83 (37.4)	156 (54.5)	382 (56.0)	35 (62.5)
Yes	590 (47.4)	139 (62.6)	130 (45.5)	300 (44.0)	21 (37.5)
**Current COVID-19 antigen-based RDT Result**					
Negative	1083 (86.9)	188 (84.7)	260 (90.9)	587 (85.1)	48 (85.7)
Positive	6 (0.5)	0 (0.0)	4 (1.4)	2 (0.3)	0 (0.0)
Declined	157 (12.6)	34 (15.32)	22 (7.7)	93 (13.6)	8 (14.3)
**Previous COVID-19 test (RDT)**					
No	330 (26.5)	80 (36.0)	68 (23.8)	173 (25.4)	9 (16.1)
Yes	916 (73.5)	142 (64.0)	218 (76.2)	509 (74.6)	47 (83.9)
**Previous COVID-19 test (PCR)**					
No	327 (26.2)	87 (39.2)	74 (25.9)	153 (22.4)	13 (23.2)
Yes	919 (73.8)	135 (60.8)	212 (74.1)	529 (77.60)	43 (76.8)
**Previously diagnosed with COVID-19 (self-reported)**					
No	1059 (85.0)	167 (75.2)	240 (84.0)	600 (88.0)	52 (92.9)
Yes	187 (15.0)	55 (24.8)	46 (16.0)	82 (12.0)	4 (7.1)
**Self-reported symptoms due to COVID-19 suspicion**					
No	923 (74.1)	129 (58.1)	218 (76.2)	530 (77.7)	46 (82.1)
Yes	323 (25.9)	93 (41.9)	68 (23.8)	152 (22.3)	10 (17.9)
**Currently on medication**					
No	925 (74.2)	185 (83.3)	223 (78.0)	470 (68.9)	47 (83.9)
Yes	321 (25.8)	37 (16.7)	63 (22.0)	212 (31.1)	9 (16.1)
***Lifestyle***					
**BMI status**					
Underweight	15 (1.2)	2 (1.0)	5 (1.8)	8 (1.2)	0 (0.0)
Normal weight	344 (27.6)	75 (33.7)	96 (33.6)	150 (21.9)	23 (41.1)
Overweight	468 (37.6)	96 (43.2)	100 (34.9)	248 (36.4)	24 (42.8)
Obese	419 (33.6)	49 (22.1)	85 (29.7)	276 (40.5)	9 (16.1)
**Alcohol intake**					
No	1123 (90.1)	186 (83.8)	245 (85.7)	644 (94.4)	48 (85.7)
Yes	123 (9.9)	36 (16.2)	41 (14.3)	38 (5.6)	8 (14.3)
**Drug use (Marijuana, Tramadol, Codeine, etc.)**					
No	1244 (99.8)	221 (99.5)	285 (99.6)	682 (100.0)	56 (100.0)
Yes	2 (0.2)	1 (0.5)	1 (0.4)	0 (0.0)	0 (0.0)
**Frequency of time spent away from work due to COVID-19 infection**					
At home for a few days	134 (10.8)	23 (10.4)	33 (11.5)	73 (10.7)	5 (9.0)
At home, most of the days	341 (27.4)	55 (24.9)	96 (33.6)	178 (26.1)	12 (21.4)
At home every day	770 (61.8)	143 (64.7)	157 (54.9)	431 (63.2)	39 (69.6)
**Knowledge of COVID-19**					
Poor	440 (35.3)	86 (38.7)	117 (40.9)	215 (31.5)	22 (39.3)
Good	806 (64.7)	136 (61.3)	169 (59.1)	467 (68.5)	34 (60.7)
**Attitude towards COVID-19 safety**					
Poor	607(48.7)	90 (40.5)	155 (54.2)	335 (49.1)	27 (48.2)
Good	639 (51.3)	132 (59.5)	131 (45.8)	347 (50.9)	29 (51.8)
**Practice of COVID-19 safety**					
Poor	1180 (94.7)	207 (93.2)	280 (97.9)	640 (93.8)	53 (94.6)
Good	66 (5.3)	15 (6.8)	6 (2.1)	42 (6.2)	3 (5.4)

*Note: BUTH: Bingham University Teaching Hospital; JUTH: Jos University Teaching Hospital; LTH: Ladoke Akintola Teaching Hospital; PSSH: Plateau State Specialist Hospital; RSUTH: Rivers State University Teaching Hospital; UCH: University College Hospital Ibadan; UPTH: University of Port Harcourt Teaching Hospital; RDT: rapid diagnostic test; PCR: polymerase chain reaction; COVID-19: coronavirus-2019; HCW: healthcare worker*

### SARS-CoV-2 IgG seropositivity by cadre of HCWs in Three Nigerian States

**Fig 1:**
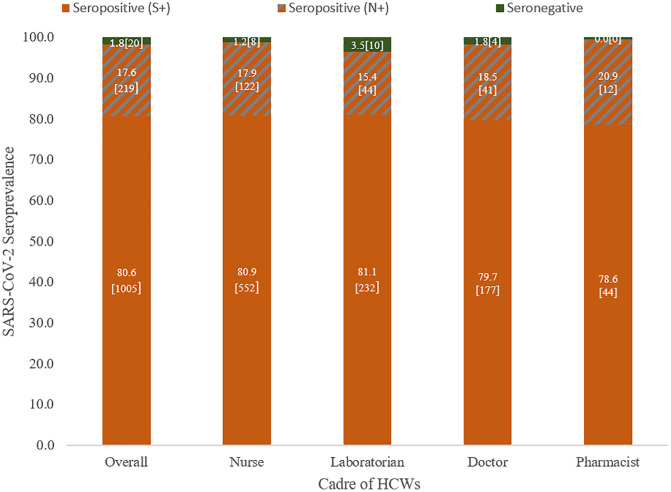
Distribution of SARS-CoV-2 IgG seropositivity by cadre of HCWs.

The SARS-CoV-2 seropositivity to S protein was 17.6% (95%CI = 15.5%−19.8%), while SARS-CoV-2 seropositivity to N protein was 80.6% (95%CI = 78.3%−82.6%). The overall SARS-CoV-2 seroprevalence [S + /RBD591 + /N+(N-)] of the HCWs in this study was 98.2%. (95%CI = 97.3%−98.8%), while SARS-CoV-2 seronegative prevalence (S-/RBD591-/N-) was only 1.8% (95%CI = 1.1%−2.7%) (Fig 1).

While SARS-CoV-2 seropositivity was high among all HCW cadres and across all the states, it was slightly lower among in Plateau State (95.8%) compared to those in Rivers (99.7%) and Oyo (99.3%) states *(p < 0.001)*. SARS-CoV-2 seropositivity was slightly higher among vaccinated HCWs (98.6%) and HCWs with travel history to COVID-19-affected areas (98.3%). SARS-CoV-2 seropositivity was lower among HCWs that tested Ag-RBD positive during enrollment (p = 0.007), slightly higher among those with satisfactory knowledge (98.6%) than those with unsatisfactory knowledge (97.5%) (S2 Table).

### Reported COVID-19 symptoms by cadre of HCWs in Three Nigerian States

Among healthcare workers (HCWs) who reported symptoms suggestive of COVID-19, the most frequently reported symptoms were body aches and pains (66.9%), tiredness (65.6%), nasal congestion (63.5%), and dry cough (60.0%). Fever was reported by 52.0% (n = 168) of symptomatic HCWs and was most common among doctors (72.0%) and pharmacists (70.0%). Additionally, loss of smell and loss of taste were reported by 40.0% and 37.8% of HCWs, respectively (S1 Table).

### Bivariate analysis of factors associated with SARS-CoV-2 IgG seropositivity among HCWs in Three Nigerian States

Table 2 shows the demographics, facility, exposure, clinical, and lifestyle factors associated with SARS-CoV-2 IgG seropositivity among HCWs at *p* < 0.20. HCW’s facility of practice was associated with N seropositivity (LR-X^2^ = 32.7, *p < 0.001*). HCW’s years of practice (LR-X^2^ = 4.43, *p = 0.035*), occupational exposure (LR-X^2^ = 4.13, *p = 0.042*), living with a person suspected with COVID-19 infection (LR-X^2^ = 4.55, *p = 0.032*) and previous COVID-19 test by RDT (LR-X^2^ = 5.61, *p = 0.018*) were associated with SARS-CoV-2 IgG seropositivity to S protein. Age, facility ownership, being tested for COVID-19 were associated antibody seropositivity to S + /RBD591 + /N- (*p < 0.10*). Also, household type and size, cadre of HCWs, blood group, and HCW on medication as well as obesity status were associated with SARS-CoV-2 IgG seropositivity based on S+ (*p < 0.20)*. Similar factors were associated with SARS-CoV-2 IgG seropositivity N protein. Facility of practice (LR-X^2^ = 14.16, *p = 0.002*), occupational exposure (LR-X^2^ = 4.20, *p = 0.040*), previous COVID-19 test by RDT (LR-X^2^ = 5.48, *p = 0.019*), and drug use (LR-X^2^ = 5.00, *p:.025*) were associated with SARS-CoV-2 IgG seropositivity based on N+ (p < 0.05). Other factors, including education, frequency of time spent away from work due to COVID-19, and knowledge of COVID-19, were associated with SARS-CoV-2 IgG seropositivity to the N protein *p < 0.20* (Table 2).

**Table 2:** Factors associated with SARS-CoV-2 seropositivity in Three Nigerian States at p < 0.20.

HCWs Characteristics	Sero+(S + /RBD591+) vs Sero-		Sero+(N+) vs Sero-		Sero+ vs Sero-	
	B (exp B)^	LR-X^2^(p-value)	B (exp B)^	LR-X^2^(p-value)	B (exp B)^	LR-X^2^(p-value)
***Demographic***						
Age	−0.46 (0.63)	**3.48 (0.062)**	−0.17 (0.84)	0.60 (0.437)	−0.22 (0.80)	0.95 (0.329)
Sex	−0.41 (0.66)	0.78 (0.377)	−0.53 (0.58)	1.42 (0.232)	−0.51 (0.59)	1.31 (0.252)
Place of residence	−0.09 (0.91)	0.02 (0.898)	−0.27 (0.76)	0.23 (0.635)	−0.25 (0.78)	0.18 (0.674)
State of residence	−0.32 (0.72)	1.53 (0.215)	0.20 (1.23)	0.54 (0.465)	0.092 (0.58)	0.12 (0.731)
Education	0.55 (1.74)	1.50 (0.220)	0.59 (1.81)	**1.86**^**d**^ **(0.173)**	0.58 (1.79)	**1.82**^**a**^ **(0.177)**
Marital status	−0.53 (0.59)	1.01 (0.313)	0.05 (1.05)	0.02 (0.902)	−0.018 (0.98)	0.01 (0.964)
Religion	−0.24 (0.79)	0.09 (0.767)	−0.64 (0.53)	0.71 (0.398)	−0.57 (0.56)	0.56 (0.456)
***Facility/Household***						
Facility of practice	−0.76 (2.14)	**32.72**^**a**^ **(<0.001)**	0.41 (1.50)	**14.16**^**b**^ **(0.002)**	0.45 (1.56)	**16.64**^**a**^ **(<0.001)**
Ownership of facility	−1.68 (0.19)	**2.81**^**c**^ **(0.093)**	−0.01 (0.99)	0.01 (0.995)	−0.17 (0.84)	0.05 (0.882)
Years of practice	−0.47 (0.62)	**4.43**^**b**^ **(0.035)**	−0.20 (0.82)	0.94 (0.331)	−0.24 (0.78)	1.40 (0.237)
Household type	−0.34 (0.71)	**2.41**^**d**^ **(0.120)**	−0.30 (0.74)	**2.00**^**d**^ **(0.157)**	−0.31 (0.73)	**2.11**^**a**^ **(0.146)**
Household size	−0.31 (0.73)	**2.55**^**d**^ **(0.110)**	−0.16 (0.85)	0.71 (0.399)	−0.19 (0.83)	0.98 (0.322)
***Exposure***						
Occupational exposure	−0.94 (0.39)	**4.13**^**b**^ **(0.042)**	−0.91 (0.40)	**4.20**^**b**^ **(0.040)**	−0.92 (0.40)	**4.26**^**b**^ **(0.039)**
COVID-19 vaccination status	0.45 (1.56)	1.00 (0.317)	0.35 (1.42)	0.66 (0.416)	0.37 (1.45)	0.73 (0.392)
Recent travel history	−0.94 (0.39)	0.56 (0.452)	−1.08 (0.34)	0.79 (0.373)	−1.05 (0.35)	0.76 (0.382)
Attended a gathering of more than 50 persons	−0.25 (0.78)	0.28 (0.599)	−0.14 (0.86)	0.10 (0.753)	−0.16 (0.85)	0.13 (0.721)
Contact with suspected COVID person	−0.01 (0.99)	0.01 (0.980)	0.01 (1.01)	0.01 (0.986)	0.01 (1.00)	0.01 (0.992)
Contact with confirmed COVID person	0.01 (1.01)	0.01 (0.988)	0.02 (1.02)	0.01 (0.957)	0.02 (1.02)	0.01 (0.963)
Live with suspected covid infected person	−2.38 (0.09)	**4.55**^**b**^ **(0.032)**	−1.50 (0.22)	**2.67**^**d**^ **(0.102)**	−1.61 (0.20)	**3.01**^**c**^ **(0.083)**
***Clinical & Health-related***						
Cadre of HCWs	0.39 (1.48)	**2.37**^**d**^ **(0.124)**	0.38 (1.46)	**2.33**^**d**^ **(0.127)**	0.38 (1.46)	**2.36**^**d**^ **(0.124)**
Blood group	0.23 (1.26)	**1.79**^**d**^ **(0.176)**	0.24 (1.27)	**2.02**^**d**^ **(0.154)**	0.24 (1.27)	**2.01**^**d**^ **(0.156)**
Ever tested for COVID-19	−0.77 (0.46)	**2.88**^**c**^ **(0.089)**	−0.65 (0.52)	**2.25**^**d**^ **(0.133)**	−0.67 (0.51)	**2.40**^**d**^ **(0.121)**
Current RDT Result	0.01 (1.00)	0.01 (0.994)	0.08 (1.09)	0.07 (0.797)	0.07 (1.07)	0.05 (0.831)
Previous COVID-19 test is by RDT	1.09 (2.98)	**5.61**^**b**^ **(0.018)**	1.03 (2.81)	**5.48**^**b**^ **(0.019)**	1.04 (2.84)	**5.61**^**b**^ **(0.018)**
Previous COVID-19 test is by PCR	0.28 (1.33)	0.33 (0.564)	0.28 (1.32)	0.34 (0.559)	0.28 (1.32)	0.35 (0.557)
Previously diagnosed with COVID-19 (self-reported)	0.42 (1.53)	0.33 (0.563)	0.61 (1.83)	0.78 (0.377)	0.58 (1.78)	0.70 (0.404)
Self-reported symptoms due to COVID-19 suspicion	−0.48 (0.62)	0.94 (0.333)	−0.25 (0.77)	0.29 (0.588)	−0.29 (0.74)	0.39 (0.533)
Currently on medication	−0.66 (0.52)	**1.84**^**d**^ **(0.174)**	−0.48 (0.62)	1.08 (0.299)	−0.51 (0.60)	1.22 (0.269)
***Lifestyle***						
BMI status (Obesity)	−0.39 (0.67)	**2.07**^**d**^ **(0.150)**	−0.36 (0.69)	**1.86**^**d**^ **(0.173)**	−0.37 (0.69)	**1.92**^**d**^ **(0.166)**
Alcohol intake	0.99 (2.71)	1.20 (0.273)	0.81 (2.24)	0.79 (0.374)	0.84 (2.32)	0.87 (0.350)
Drug use (Marijuana, Tramadol, Codeine)	---------	---------	−3.86 (0.02)	**5.00**^**b**^ **(0.025)**	−4.06 (0.02)	**5.38**^**b**^ **(0.020)**
Frequency of time spent away from work due to COVID-19	0.38 (1.46)	1.55 (0.213)	0.45 (1.56)	**2.24**^**d**^ **(0.135)**	0.43 (1.54)	**2.12**^**d**^ **(0.145)**
Knowledge of COVID-19	0.42 (1.52)	0.86 (0.353)	0.66 (1.94)	**2.31**^**d**^ **(0.128)**	0.62 (1.85)	**2.02**^**d**^ **(0.155)**
Altitude towards COVID-19 safety	−0.05 (0.95)	0.01 (0.919)	−0.15 (0.86)	0.12 (0.724)	−0.13 (0.87)	0.10 (0.757)
Practice of COVID-19 safety	0.28 (1.33)	0.08 (0.783)	0.14 (1.15)	0.02 (0.893)	0.16 (1.18)	0.03 (0.871)

**Note:** ^**a**^**Significant at p < 0.001;** ^**b**^**Significant at p < 0.05;** ^**c**^**Significant at p < 0.010;** ^**d**^**Significant at p < 0.20; LR-X**^**2**^ **Likelihood Ratio Chi-square; B Regression Coefficient; (exp B) exponentiated Regression Coefficient; ^Estimate of Bivariate Log-binomial Regression; sero += seropositive; sero- = seronegative.**

### Multivariate logistic regression analysis of factors associated with SAR-CoV-2 IgG seropositivity among HCWs in Three Nigerian States

The multivariable logistic regression explored in model blocks for each SARS-CoV-2 IgG seropositive natural (previous/current), and all infection versus seronegative is presented in Table 3. HCWs that were occupationally exposed to SARS-CoV-2 had a 96% lower risk of seropositivity from previous infection compared to their occupationally unexposed counterpart (aRR = 0.04, 95%CI = 0.004–0.58). Nurses were more likely to be seropositive due to natural infection than laboratorians (aRR = 386.33, 95%CI= (9.34–15978.8). Odds of seropositivity were reduced by 93% in HCWs with the A blood group compared to the O blood group. The likelihood of seropositivity due to previous infection was significantly higher among HCWs practicing at University of Port Harcourt Teaching Hospital (UPTH) compared to BUTH (aRR = 442.47, 95%CI = 3.59–54434.99**, p** *< .05*). The odds of SARS-CoV-2 IgG seropositivity due to previous infection were also higher among HCWs with 6–10 years working experience compared to those with >15 years’ experience. HCWs within age-group of 51–60 years compared to those in age-group 21–30 years had higher odds of SARS-CoV-2 IgG seropositivity. After adjusting for other factors, nurses’ odds of SARS-CoV-2 IgG seropositivity were three times higher than laboratorians (aRR = 3.02, 95%CI = 1.10–9.05) (Table 3).

**Table 3:** Multivariate logistic regression analysis of factors associated with SAR-CoV-2 IgG seropositivity among HCWs in Three Nigerian States.

HCWs Characteristics	Sero+(S + /RBD591+) vs Sero-		Sero+(N+) vs Sero-		Sero+ vs Sero-	
**cRR (95%CI)**	**aRR (95%CI)**	**cRR (95%CI)**	**aRR (95%CI)**	**cRR (95%CI)**	**aRR (95%CI)**
***Demographic***						
**Age**						
21–30 years	Ref	Ref				
31–40 years	0.93 (0.10-8.13)	244.03 (0.05-1055329)^				
41–50 years	0.67 (0.07-6.06)	----				
51–60 years	0.42 (0.04-3.77)	**----**				
>60 years	----------	**----------**				
**Education**						
Tertiary			1.80 (0.77-4.21)	1.41 (0.49- 4.06)	1.79 (0.77-4.17)	1.54 (0.53-4.38)
Postgraduate			Ref	Ref	Ref	Ref
***Facility/Household***						
**Facility of practice**						
BUTH	Ref	Ref	Ref	Ref	Ref	ref
JUTH	1.05 (0.16-6.72)	14.28 (0.32- 628.94)	0.40 (0.08-1.90)	0.49 (0.08- 2.95)	0.43 (0.09-2.03)	0.52 (0.08-3.09)
LTH	3.99 (0.50-31.9)	**----**	0.51 (0.08- 3.15)	0.36 (0.04-2.96)	0.66 (0.10-4.04)	0.53 (0.06-4.15)
PSSH	0.92 (0.12-6.56)	**----**	0.36 (0.07- 1.84)	0.50 (0.08- 2.97)	0.38 (0.07-1.96)	0.52 (0.08-3.09)
RSUTH	-----------	----------	----------	----------	----------	--------
UCH	-----------	----------	-----------	----------	----------	--------
UPTH	14.5 (1.05-198)*	442.47 (3.59 −54434.99)* ^	4.79 (0.42- 53.50)	4.52 (0.34- 59.70)	5.2 (0.46-58.02)	4.98 (0.37-66.01)
**Years of practice**						
< 2 years	1.80 (0.20-15.5)	102.12 (0.03-272313.5) ^				
2-5 years	3.79 (0.46-31.1)	----				
6-10 years	5.32 (1.13-25.0)*	**-----**				
11-15 years	1.75 (0.63-4.80)	33.73 (0.45- 2487.09) ^				
>15 years	Ref	Ref				
**Household type**						
Self-contain	Ref	Ref	Ref	Ref	Ref	Ref
1 bedroom	2.83 (0.26-29.9)	0.99 (0.0006- 1548.26)	3.5 (0.35- 34.39)	**-------------**	3.36 (0.34-32.95)	-------
2 bedrooms	2.63 (0.53-12.8)	3.49 (0.02-512.56)	2.45 (0.53- 11.24)	2.16 (0.40-11.57)	2.49 (0.54-11.33)	1.88 (0.35-9.94)
3 bedrooms	1.63 (0.40-6.52)	15.87 (0.23-1077.04)	1.98 (0.53-7.38)	3.65 (0.80-16.56)	1.91 (0.51-7.08)	3.54 (0.78-16.06)
Other	0.96 (0.18-4.84)	5.55 (0.05- 557.07)	1.42 (0.31-6.55)	2.27 (0.38-13.56)	1.33 (0.29-6.09)	1.99 (0.34-11.65)
**Household size**						
1	Ref	Ref				
2	------------	-----------				
3	0.55 (0.05-5.34)	0.001 (5.00e-08–23.76)				
4	0.92 (0.08-9.45)	0.04 (3.42e-06–544.05)				
5+	0.49 (0.06-4.04)	0.03 (3.86e-06–260.02)				
***Exposure***						
**Occupational exposure**						
Unexposed	Ref	Ref	Ref	Ref	Ref	Ref
Exposed	0.39 (0.15-0.99)*	0.04 (0.004- 0.58)*	0.40 (0.16-0.99)*	0.53 (0.18-1.55)	0.40 (0.16-0.98)*	0.50 (0.17-1.46)
**Live with suspected covid infected person**						
No	Ref	Ref	Ref	Ref	Ref	Ref
Yes	0.09 (0.01-0.69)*	0.52 (0.00001- 17436.41)	0.22 (0.04- 1.02)	0.43 (0.07-2.54)	0.20 (0.04-0.90)*	0.40 (0.07-2.34)
***Clinical & Health-related***						
**Cadre of HCWs**						
Laboratorians	Ref	Ref	Ref	Ref	Ref	Ref
Doctors	2.33 (0.67-8.01)	48.81 (1.68- 1417.23)*	1.90 (0.58- 6.18)	3.04 (0.63-14.56)	1.97 (0.61-6.38)	3.35 (0.70-16.01)
Nurses	3.46 (1.28-9.34)*	386.33 (9.34- 15978.8)*^	2.97 (1.15-7.63)*	3.02 (1.10-9.05)*	3.05 (1.19-7.82)*	3.20 (1.08-9.45)*
Pharmacist	----------	------------	----------	----------	---------	--------
**Blood group**						
A	0.41 (0.14-1.19)	0.07 (0.01-0.99)*	0.39 (0.14- 1.10)	0.48 (0.15-1.54)	0.39 (0.14-1.10)	0.48 (0.15-1.55)
B	0.35 (0.11-1.07)	0.04 (0.002- 0.73)*	0.38 (0.13- 1.10)	0.87 (0.23-3.22)	0.37 (0.13-1.08)	0.84 (0.22-3.09)
AB	-----------	------------	----------	----------	---------	------
O	Ref	Ref	Ref	Ref	Ref	Ref
**Ever tested for COVID-19**						
No	Ref	Ref	Ref	Ref	Ref	Ref
Yes	0.46 (0.18-1.15)	0.84 (0.03-23.62)	0.51 (0.21-1.25)	1.43 (0.33-6.12)	0.50 (0.21-1.22)	1.48 (0.35-6.30)
**Previous COVID-19 test is by RDT**						
No	Ref	Ref	Ref	Ref	Ref	Ref
Yes	0.34 (0.13-0.82)*	0. 47 (0.02-10.94)	0.35 (0.15-0.83)	0.63 (0.15-2.59)	0.35 (0.15-0.82)*	1.47 (0.36-5.98)
**Currently on medication**						
No	Ref	Ref				
Yes	0.52 (0.20-1.30)	1.64 (0.20-12.91)				
***Lifestyle***						
**BMI status (Obesity)**						
Underweight			Ref	Ref	Ref	Ref
Normal weight			2.70 (0.74- 9.78)	2.78 (0.63-12.19)	2.74 (0.75-9.88)	2.65 (0.60-11.54)
Overweight			-----------	-----------	---------	-------
Obese			1.30 (0.51-3.28)	0.87 (0.29-2.2.55)	1.24 (0.49-3.10)	0.89 (0.30-2.61)
**Drug use (Marijuana, Tramadol, Codeine, etc.)**						
No			Ref	Ref	Ref	Ref
Yes			0.02 (0.001-0.35)*	0.04 (0.001-1.16)	0.02 (0.001-0.28)**	0.04 (0.001-0.95)*
**Frequency of time spent away from work due to COVID-19**						
At home a few of the days			Ref	Ref	Ref	Ref
At home most of the days			1.50 (0.31-7.18)	0.90 (0.14-5.59)	1.44 (0.30-6.87)	0.25 (0.04-1.43)
At home every day			0.48 (0.10- 2.21)	0.25 (0.04-1.48)	0.45 (0.09-2.08)	0.84 (0.13-5.05)
**Knowledge of COVID-19**						
Poor			Ref	Ref	Ref	Ref
Good			1.93 (0.83-4.52)	1.64 (0.61-4.41)	1.85 (0.79-4.31)	1.62 (0.60-4.30)

Note: **significant at p < 0.01; *significant at p < 0.05; ^adjusted estimate higher than 100 with wide interval due to small sample; cRR Crude Odds Ratio; aRR Adjusted Odds Ratio; CI Confidence Interval.

## Discussion

In this study, we assessed the seroprevalence of SARS-CoV-2 among healthcare workers (HCWs) across multiple health facilities and observed a high prevalence of SARS-CoV-2 IgG antibodies between August 2022 through December 2022. In contrast, a similar study conducted in Lagos during the early phase of the pandemic (December 2020 to July 2021) reported a lower seroprevalence of 30.9% [4]. Comparable findings were reported in another study, which documented a SARS-CoV-2 seroprevalence of 72.4% among HCWs in early 2021 [16]. Our study was conducted after the fourth pandemic wave where new variants such as delta (2021) and omicron (2022) were identified, providing more time for possible exposure among HCWs in Nigeria. Additionally, the scale up vaccination may have influenced the high seropositivity reported as approximately 50% of the HCWs were vaccinated prior to the study. This may have accounted for the higher seroprevalence of COVID-19 among HCWs. A previous study on HCWs indicated an increasing trend in SARS-CoV-2 seropositivity from 31% at baseline to 45% after three months and 70% after six months of follow-up [17].

Studies on pre-pandemic samples have been conducted in Nigeria, Gabon, and Senegal reporting data on cross-reactive humoral responses against SARS-CoV-2 S and N glycoproteins on pre-pandemic samples [22,23]. However, one of these studies used an ELISA based diagnostic method and found that these cross-reactive antibodies had no significant reduction on the SARS-CoV-2 viral load of lungs [24]. A previous study showed higher recognition of human coronaviruses in Tanzania and Zambia pre-pandemic samples than in the U.S [24]. These may suggest pre-existing humoral immunity distinct to sub-Saharan African populations. Another study from Mali in 2021 showed high SARS-CoV-2 seropositivity (61.8%) amongst frontline HCWs [25] and in a systematic review reported high SARS-CoV-2 seroprevalence in the African population, suggestive of high exposure to the virus and a potential for population immunity and lesser risk for severe diseases [26]. Our study adopted several multivariate logistic regression analyses in five model blocks. In model blocks IV and V, nurses were more likely to be seropositive for COVID-19 than laboratorians. Our findings are similar to those of other studies in the U.S. and Egypt that reported higher odds of COVID-19 seropositivity among nurses, with doctors as the reference cadre [27,28]. Nurses may have higher occupational exposure to COVID-19 infected patients with longer contact times during care and treatment making them have an increased risk of exposure and infection within the cadre compared to other HCWs.

We assessed the association between knowledge, attitudes, and practices (KAP) and SARS-CoV-2 seroprevalence, which, to the best of our knowledge, is the first such analysis conducted in a Nigerian population. A study conducted across eleven Nigerian states found that community healthcare workers (HCWs) demonstrated high levels of KAP, as well as a strong capacity to provide health education and promote adherence to national COVID-19 guidelines. [29]. Although most HCWs demonstrated good knowledge of COVID-19, this did not translate into appropriate IPC practices. Similar finding were reported by Harun et al [30]. These findings underscore the need for interventions that not only improve knowledge but also strengthen adherence to infection prevention and control measures and promote behavior change among healthcare workers [31]. Overall, our findings indicate that workplace-related factors, particularly occupational exposure and healthcare facility of practice, were the strongest correlates of SARS-CoV-2 seropositivity among healthcare workers. Strengthening IPC programmes in clinical sites, ensuring consistent access to appropriate personal protective equipment, and implementing facility-specific risk mitigation strategies remain critical.

A recent study following vaccination trends in the U.S. shows that COVID-19 vaccination reduced COVID-19-related cases and hospitalizations [32]. Vaccination is the most effective public health intervention and the cornerstone of the most cost-effective healthcare interventions, and addressing vaccine hesitancy is crucial to lessen the negative consequences of the COVID-19 [33,34]. Despite the availability of COVID-19 vaccines, vaccination coverage among HCWs in our study remained suboptimal. Previous studies in Nigeria have identified educational level, occupation, religion, and prior COVID-19 diagnosis as key determinants of vaccine uptake. [35].

We had some limitations in our study. The study was conducted in three states of the country which may not be representative of all HCWs in Nigeria. The study population was predominantly composed of experienced healthcare workers employed in urban public health facilities. The high COVID-19 seropositivity of HCWs may have introduced bias in the comparison between groups, either by masking variation in disease exposure or the level of immunity. We also did not differentiate seropositivity due to natural infection or COVID-19 vaccination. The scale-up of COVID-19 vaccines amongst HCWs and across the population may have also accounted for the higher seropositivity rate, as they were the first group to be vaccinated in Nigeria. We also do not have data on full or partial COVID-19 vaccinations received by HCWs in our study which limited our ability to determine if the detection of antibodies was due to natural infection or vaccination. However, this is a multi-state study and provides data on a larger proportion of HCWs compared to other studies conducted in one state. We are also the first to assess factors responsible for COVID-19 infection among HCWs using five model blocks. Thus, the statistical model incorporated in this study accurately assesses the factors responsible for SARS-CoV-2 seropositivity while considering potential confounding variables.

Our analysis indicates that HCWs’ attitudes and practices towards COVID-19 were relatively poor, and KAP was not associated with COVID-19 infection. Given the role of KAP in lowering the risk of infectious diseases, interventions towards enhancing HCWs’ attitude and practices of HCWs to infectious diseases and prevention strategies may be beneficial in ensuring disease prevention and control measures during disease outbreaks and pandemics. Also, interventions like infection control training, supply of personal protective equipment and surveillance testing are important in mitigating the risk of the seropositivity amongst high-risk groups.

## Supporting information

S1 File Questionnaire for Knowledge Attitudes and Practices of COVID-19 among Healthcare Workers in Nigeria. (PDF)

S1 Table Self-reported Symptoms by Cadre HCWs. (PDF)
