# Supplementary material for: High prevalence of SARS-CoV-2 antibodies among frontline healthcare workers during the COVID-19 pandemic in three states of Nigeria from August to December 2022
Source: PLoS One. 2026 Aug 10;21(8):e0354606. doi: 10.1371/journal.pone.0354606 (PMC13456288; doi:10.1371/journal.pone.0354606)
Supplement: Supplementary file 1 [file pone.0354606.s001.pdf]

S1 File: Questionnaire for Knowledge Attitudes and Practices of COVID-19 among Healthcare Workers in Nigeria

KAP-SURVEY	
**SECTION A: SOCIO-DEMOGRAPHICS**	
1. Place of Residence:	☐ Rural<br>☐ Semi-urban<br>☐ Urban
2. LGA of Residence:	____________________
3. State of Residence:	____________________
4. Type of facility	☐ Private<br>☐ Government owned/Public<br>☐ Other, please specify _________
5. Level of health facility	☐ Primary<br>☐ Secondary<br>☐ Tertiary
6. Gender:	Male ☐<br>Female ☐<br>Others; Please specify ________________________
7. Cadre of Healthcare worker	Doctor ☐ Nurse ☐ Pharmacist ☐ Lab Scientist ☐<br>Other, please specify _________________
8. Years of practice or experience	☐ < 2<br>☐ 2- 5years<br>☐ 6 - 10years<br>☐ 11-15years<br>☐ >15 years
9. Date of Birth	
10. Age (years)	☐ <20 years ☐ 41-50 years<br>☐ 21-30 years ☐ 51-60 years<br>☐ 31-40 years ☐ >60 years
10. Marital Status:	Single ☐ Married ☐ Divorced ☐ Separated ☐<br>Widow ☐ Widower ☐ Cohabiting ☐
11. Religion:	Christianity ☐ Islam ☐ Traditional Religion ☐<br>Other; Please specify __________________________
12. Nationality:	Nigerian ☐ Foreigner ☐
If foreigner, please specify nationality:	________________________
13. Highest Education Level	Primary ☐ Secondary ☐ Tertiary Postgraduate ☐
14. Blood Group	A ☐ AB ☐ B ☐ O ☐<br>☐ Rhesus+ ☐ Rhesus-
15. Does your facility provide care and treatment for patients with COVID-19?	Yes ☐ No ☐

15b. If yes, how often do you provide care or have been in contact with a suspected or confirmed COVID-19 case(s)	☐ Never 0 Day/week<br>☐ Rarely 1 day/week<br>☐ Occassionally 2 days/week<br>☐ Sometimes 3 days/week<br>☐ Frequently 4 days/week<br>☐ Always >5 days/week
16. Do you provide care or treatment to a suspected or confirmed COVID-19 case at your facility?	Yes ☐ No ☐
17. Since the pandemic, how many suspected or confirmed COVID-19 patients did you care for or come in contact with at work?	______________________________
18. Since the COVID-19 pandemic, how long (in days) did you care for or were you in contact with a suspected or confirmed COVID19 case?	______________________________
19. Which hospital unit do you work in and for how long?	______________________________
20. What is your primary source of information on COVID-19 (tick all that applies)	☐ International Organization<br>☐ Government websites and media<br>☐ News Media such as radio, tv, newspaper<br>☐ Social media such as Whatsapp, facebook etc.<br>☐ Health facility<br>☐ Journals<br>Others, please specify ____________________

SECTION B: KNOWLEDGE

21. The Main clinical symptoms of COVID-19 are (Tick all that applies)
Dry persistent cough ☐
Fever ☐
Sore throat ☐
Runny nose ☐
Shortness of breath ☐
Myalgia (muscle pain) ☐
Diarrhea ☐

Please select most appropriate option

22. Clinical management and treatment for COVID-19 cases are focused on Infection prevention and control and supportive care for common symptoms and complications and as well Clinical monitoring	Yes ☐ No ☐ I don't know ☐
23. Eating or encountering wild animals would result in the infection by COVID-19 virus	Yes ☐ No ☐ I don't know ☐
24. Symptoms of common cold, stuffy nose, running and sneezing are common with all persons infected with the COVID-19"	Yes ☐ No ☐ I don't know ☐
25. Persons with COVID-19 cannot transmit the virus to others when signs and symptoms are not present	Yes ☐ No ☐ I don't know ☐
26. The COVID-19 virus spreads via respiratory droplets of infected individuals	Yes ☐ No ☐ I don't Know ☐
27. Wearing non-medical masks can prevent one from acquiring infection by the COVID-19 virus	Yes ☐ No ☐ I don't know ☐
28. It is not necessary for children and young adults to take measures to prevent the infection of COVID-19 virus	Yes ☐ No ☐ I don't know ☐
29. To prevent the infection of COVID-19, individuals should avoid going to crowded places such as social gatherings, religious, bus parks and avoid taking public transportations	Yes ☐ No ☐ I don't know ☐
30. Isolation and treatment of people who are infected with COVID-19 virus are effective ways to reduce the spread of the virus	Yes ☐ No ☐ I don't know ☐

31. People who have contact with confirmed COVID-19 cases should immediately be in self-isolation or observation for a period of 14 days	Yes ☐ No ☐ I don't know ☐
32. Any individual with a history of contact with a confirmed or probable COVID-19 case in the last 14 days prior to symptom onset is a suspected case	Yes ☐ No ☐ I don't know ☐
33. A person with laboratory confirmation of COVID-19 infection, irrespective of clinical signs and symptoms is a confirmed case	Yes ☐ No ☐ I don't know ☐
34. The incubation period of COVID-19 is 2-14 days	Yes ☐ No ☐ I don't know ☐
35. Not all patients infected with COVID-19 will develop severe illness.	Yes ☐ No. ☐ I don't know ☐
36. Patients with underlying chronic disease conditions are at higher risk of infection and death from COVID-19.	Yes☐ No ☐ I don't know ☐
37. The elderly are at higher risk of Severe COVID-19 disease	Yes☐ No ☐ I don't know ☐
38. Pregnant are at higher risk of Severe COVID-19 disease	Yes☐ No ☐ I don't know ☐
39. Antibiotics are the first line of treatment when you suspect or have a confirmed case of COVID-19	Yes ☐ No ☐ I don't know ☐
40. PPE and respiratory protection cannot effectively protect the users if it is not properly and consistently worn	Yes ☐ No ☐ I don't know ☐
41. N95 filtering face piece respirators (FFRs) are designed to protect the wearer and surgical masks are designed to protect the patient	Yes ☐ No ☐ I don't know ☐
42. The COVID-19 Vaccine can confer protection on everyone	Yes☐ No ☐ I don't know ☐
43. Are you aware of any type of COVID-19 vaccination?	Yes ☐ No ☐ I don't know ☐
43b. If yes, (tick all that apply)	☐ Astrazeneca<br>☐ Pfizer<br>☐ Moderna<br>☐ Sputnik<br>☐ Johnson and Johnson<br>Other please specify____________________
**SECTION C: ATTITUDES**	
44. Black Race is protective towards COVID-19 disease	Strongly Agree ☐<br>Agree ☐<br>Neutral ☐<br>Strongly diagree ☐<br>Disagree ☐
45. Participating in community sensitization on COVID-19 will help reduce the spread of the virus	Strongly Agree ☐<br>Agree ☐<br>Neutral ☐<br>Strongly diagree ☐

	Disagree ☐
46. Health insurance or incentives can motivate healthcare workers directly involved in the management of COVID-19 patients	Strongly Agree ☐<br>Agree ☐<br>Neutral ☐<br>Strongly diagree ☐<br>Disagree ☐
47. Wearing a well-fitting face mask is effective in preventing COVID-19	Strongly Agree ☐<br>Agree ☐<br>Neutral ☐<br>Strongly diagree ☐<br>Disagree ☐
48. Effective hand washing (washing for at least 30 seconds) can prevent you from getting COVID-19	Strongly Agree ☐<br>Agree ☐<br>Neutral ☐<br>Strongly diagree ☐<br>Disagree ☐
49. When a patient has signs and symptoms of COVID-19, I can confidently participate in the management of the patient	Strongly Agree ☐<br>Agree ☐<br>Neutral ☐<br>Strongly diagree ☐<br>Disagree ☐
50. COVID-19 vaccine recommendation reduce/stop the spread of COVID-19 disease	Strongly Agree ☐<br>Agree ☐<br>Neutral ☐<br>Strongly diagree ☐<br>Disagree ☐
51. Since the COVID-19 pandemic, how often were you fearful on contracting the virus?	☐ Almost everytime<br>☐ Everytime<br>☐ Occassionally
52. How often do you worry about yourself and/or your loved ones	☐ Almost everytime<br>☐ Everytime<br>☐ Occassionally
**SECTION D: PRACTICES**	
53. How often have you gone to crowded places?	☐ Never<br>☐ Rarely (in <10% of the chances when I could have<br>☐ Ocassionally (In about 30% of the chances when I could have)<br>☐ Sometimes (In about 3<br>50% of the chances when I could have)<br>☐ Frequently (In about 70% of the chances when I could have)<br>☐ Usually (In about 90% of the chances when I could have)<br>☐ Everytime
54. How often do you wear masks when in contact with people/patients?	☐ Never<br>☐ Rarely (in <10% of the chances when I could have<br>☐ Ocassionally (In about 30% of the chances when I could have)<br>☐ Sometimes (In about 3<br>50% of the chances when I could have)<br>☐ Frequently (In about 70% of the chances when I could have)<br>☐ Usually (In about 90% of the chances when I could have)<br>☐ Everytime

55a. What type of mask do you use regularly within the hospital/facility	☐ N95<br>☐ KN95<br>☐ Surgical mask<br>☐ Fabric mask<br>☐ Faceshield
55b. What type of mask do you use regularly outside the hospital/facility	☐ N95<br>☐ KN95<br>☐ Surgical mask<br>☐ Fabric mask<br>☐ Faceshield
56. How often do you effectively wash (washing for at least 30 seconds) your hands before and after patients or encountering people	☐ Never<br>☐ Rarely (in <10% of the chances when I could have<br>☐ Ocassionally (In about 30% of the chances when I could have)<br>☐ Sometimes (In about 3 50% of the chances when I could have)<br>☐ Frequently (In about 70% of the chances when I could have)<br>☐ Usually (In about 90% of the chances when I could have)<br>☐ Everytime
57. How often do you avoid patients with signs and symptoms suggestive of COVID-19	☐ Never<br>☐ Rarely (in <10% of the chances when I could have<br>☐ Ocassionally (In about 30% of the chances when I could have)<br>☐ Sometimes (In about 3 50% of the chances when I could have)<br>☐ Frequently (In about 70% of the chances when I could have)<br>☐ Usually (In about 90% of the chances when I could have)<br>☐ Everytime
58. How often do you use non-conventional remedies (honey, garlic, and lime) when you have flu-like symptoms	☐ Never<br>☐ Rarely (in <10% of the chances when I could have<br>☐ Ocassionally (In about 30% of the chances when I could have)<br>☐ Sometimes (In about 3 50% of the chances when I could have)<br>☐ Frequently (In about 70% of the chances when I could have)<br>☐ Usually (In about 90% of the chances when I could have)<br>☐ Everytime
