# Supplementary material for: High prevalence of SARS-CoV-2 antibodies among frontline healthcare workers during the COVID-19 pandemic in three states of Nigeria from August to December 2022
Source: PLoS One. 2026 Aug 10;21(8):e0354606. doi: 10.1371/journal.pone.0354606 (PMC13456288; doi:10.1371/journal.pone.0354606)
Supplement: Supplementary file 2 [file pone.0354606.s002.pdf]

S1 Table: Self-reported Symptoms by Cadre HCWs

Symptoms	Overall 323 (100) n (%)	Doctors 93 (28.8) n (%)	Laboratorians 68 (21.1) n (%)	Nurses 152 (47.1) n (%)	Pharmacist 10 (3.1) n (%)	p-value
**Dry cough**						0.280
No	110(34)	26(28.0)	28(41.2)	54(36.0)	2(20.0)	
Yes	194(60)	63(67.7)	34(50.0)	89(58.6)	8(80.0)	
Missing	19(6.0)	4(4.3)	6(8.8)	9(5.9)	0(0.0)	
**Fever**						0.481
No	135(41.8)	34(36.6)	33(48.5)	64(42.1)	4(40.0)	
Yes	168(52.0)	55(59.1)	29(42.7)	78(51.3)	6(60.0)	
Missing	20(6.1)	4(4.3)	6(8.8)	10(6.6)	0(0.0)	
**Tiredness**						0.675
No	87(26.9)	20(21.5)	19(27.9)	45(29.6)	3(30.0)	
Yes	212(65.6)	67(72.0)	42(61.8)	96(63.1)	7(70.0)	
Missing	24(7.5)	6(6.5)	7(10.3)	11(7.3)	0(0.0)	
**Conjunctivitis**						0.002
No	271(83.9)	73(78.4)	59(86.7)	132(86.8)	7(70.0)	
Yes	14(4.3)	6(6.5)	1(1.5)	4(2.7)	3(30.0)	
Missing	38(11.8)	14(15.1)	8(11.8)	16(10.5)	0(0.0)	
**Loss of smell**						0.581
No	171(52.9)	47(50.5)	36(52.9)	83(54.6)	5(50.0)	
Yes	128(39.6)	38(40.9)	30(44.1)	55(36.2)	5(50.0)	
Missing	24(7.5)	8(8.6)	2(3.0)	14(9.2)	0(0.0)	
**Loss of taste**						0.006
No	176(54.5)	62(66.60)	29(42.7)	78(51.3)	7(70.0)	
Yes	122(37.8)	21(22.6)	36(52.9)	62(40.8)	3(30.0)	
Missing	25(7.7)	10(10.8)	3(4.4)	12(7.9)	0(0.0)	
**Altered Consciousness**						0.691
No	276(85.4)	77(82.8)	58(85.3)	131(86.2)	10(100)	
Yes	7(2.2)	1(1.1)	2(2.9)	4(2.6)	0(0.0)	
Missing	40(12.4)	15(16.1)	8(11.8)	17(11.2)	0(0.0)	
**Nasal congestion**						0.073
No	102(31.5)	22(23.6)	20(29.4)	58(38.2)	2(20.0)	
Yes	205(63.5)	68(73.1)	46(67.6)	83(54.6)	8(80.0)	
Missing	16(5.0)	3(3.3)	2(3.0)	11(7.2)	0(0.0)	
**Aches & pains**						0.258
No	85(26.3)	19(20.4)	23(33.8)	42(27.6)	1(10.0)	
Yes	216(66.9)	69(74.2)	39(57.4)	99(65.1)	9(90.0)	
Missing	22(6.8)	5(5.4)	6(8.8)	11(7.3)	0(0.0)	
**Diarrhea**						0.615
No	254(78.6)	72(77.4)	55(80.9)	118(77.6)	9(90.0)	
Yes	30(9.3)	6(6.5)	7(10.3)	16(10.6)	1(10.0)	
Missing	39(12.1)	15(16.1)	6(8.8)	18(11.8)	0(0.0)	
**Runny nose**						0.343
No	130(40.3)	29(31.2)	29(42.6)	68(44.7)	4(40.0)	
Yes	169(52.3)	58(62.3)	34(50.0)	71(46.7)	6(60.0)	
Missing	24(7.4)	6(6.5)	5(7.4)	13(8.6)	0(0.00	

**Difficulty breathing**						0.123
No	231(71.5)	67(72.0)	45(66.2)	113(74.3)	6(60.0)	
Yes	58(17.9)	12(12.9)	17(25.0)	25(16.5)	4(40.0)	
Missing	34(10.6)	14(15.1)	6(8.8)	14(9.2)	0(0.0	
**Sore throat**						0.082
No	137(42.4)	31(33.3)	36(52.9)	68(44.7)	2(20.0)	
Yes	157(48.6)	51(54.8)	26(38.2)	72(47.4)	8(80.0)	
Missing	29(9.0)	11(11.9)	6(8.9)	12(7.9)	0(0.0)	
**Other**						0.048
No	226(70.0)	67(72.0)	54(79.4)	96(63.2)	9(90.0)	
Yes	24(7.4)	10(10.8)	4(5.9)	10(6.5)	0(0.0)	
Missing	73(22.6)	16(17.2)	10(14.7)	46(30.3)	1(10.0)	

Note: n: number; %: percent
